# Characterizing the circularly oriented macular pigment using spatiotemporal sensitivity to structured light entoptic phenomena

**DOI:** 10.1167/jov.25.6.11

**Published:** 2025-05-28

**Authors:** Dmitry A. Pushin, Davis V. Garrad, Connor Kapahi, Andrew E. Silva, Pinki Chahal, David G. Cory, Mukhit Kulmaganbetov, Iman Salehi, Melanie A. Mungalsingh, Taranjit Singh, Benjamin Thompson, Daniel Yu, Dusan Sarenac

**Affiliations:** 1 Institute for Quantum Computing, University of Waterloo, Ontario, Canada; 2 Department of Physics and Astronomy, University of Waterloo, Ontario, Canada; 3 Centre for Eye and Vision Research, 17W Hong Kong Science Park, Hong Kong; 4 Incoherent Vision Inc., Wellesly, Ontario, Canada; 5 School of Optometry and Vision Science, University of Waterloo, Waterloo, Ontario, Canada; 6 Department of Physics, University at Buffalo, State University of New York, Buffalo, New York, USA; 7 Department of Chemistry, University of Waterloo, Waterloo, Ontario, Canada; 8 David R. Cheriton School of Computer Science, University of Waterloo, Waterloo, Ontario, Canada; 9 Glaucoma Department, Kazakh Eye Research Institute, Almaty, Kazakhstan; 10 Institute for Quantum Computing, University of Waterloo, Ontario, Canada

**Keywords:** circularly oriented macular pigment optical density, age-related macular degeneration, psychophysics, biomarkers, macular degeneration

## Abstract

To characterize the optical density of circularly oriented macular pigment (MP) in the human retina, as a quantification of macular health, Psychophysical discrimination tests were performed on human subjects using structured light-induced entoptic phenomena. Central exclusions were used to determine the visual extents of stimuli with varying spatiotemporal frequencies. A model was developed to describe the action of circularly oriented MP, and map stimuli to perceived sizes. The experimental results provided validation for the computational model, showing good agreement between measured data and predictions with a Pearson χ^2^ fit statistic of 0.06. This article contains a description of a new quantification of macular health and the necessary tools for clinical development. The integration of structured light into vision science has led to the development of more selective and versatile entoptic probes of eye health that provide interpretable thresholds of structured light perception. This work develops a model that maps perceptual thresholds of entoptic phenomena to the underlying MP structure that supports its perception. We selectively characterize the circularly oriented MP optical density, rather than the total MP optical density as typically measured. The presented techniques can be applied in novel early diagnostic tests for a variety of diseases related to macular degeneration such as age-related macular degeneration, macular telangiectasia, and pathological myopia. This work both provides insights into the microstructure of the human retina and uncovers a new quantification of macular health.

## Introduction

Xanthophylls lutein, zeaxanthin, and meso-zeaxanthin constitute the macular pigment (MP) in the retina that provides protection from the accumulation of photochemical damage (Boulton, Różanowska, & Różanowski, 2001). A portion of the MP is in a layer of radially oriented Henle fibers, where the MP is on average oriented perpendicularly to the Henle fibers (Grudzinski et al., 2017). We refer to this subset of MP as the circularly oriented MP (coMP). The effects arising from this arrangement as well as MP’s inherent dichroism and absorption spectrum, which peaks with wavelengths around 450 nm, combine to act as a weak radial polarization filter in the eye for blue light (Horváth & Varju, 2004). Thus, a faint entoptic phenomenon known as the Haidinger’s brush appears within approximately 2° retinal eccentricity when uniformly polarized blue light is viewed (Haidinger, 1844; Misson, Temple, & Anderson, 2020; Mottes, Ortolan, & Ruffato, 2022; O’Shea, Misson, & Temple, 2021). The size of polarization-related entoptic phenomena can be enhanced to approximately 5° retinal eccentricity through the use of structured light (Bliokh et al., 2023; Kapahi et al., 2024). Further, whereas Haidinger’s brush traditionally contains two azimuthal fringes, structured light-based stimuli can be constructed to produce percepts with arbitrary numbers of fringes and occlusions to probe polarized light perception with greater detail (Pushin, Cory, Kapahi, Kulmaganbetov, & Mungalsingh, 2023; Pushin, Kapahi, Silva, Cory, & Kulmaganbetov, 2024; Sarenac et al., 2020; Sarenac et al., 2022).

Low concentrations of MP have been linked to a higher risk of developing age-related macular degeneration (AMD) (Bernstein et al., 2002; Kar et al., 2020; Landrum, Bonet, & Kilburn, 1996; Ozyurt et al., 2017; Scripsema, Hu, & Rosen, 2015; Wu, Cho, Willett, Sastry, & Schaumberg, 2015). AMD is a globally leading cause of irreversible blindness (Thomas, Mirza, & Gill, 2021) that is often left undiagnosed until after the disease has progressed substantially and visual impairment has begun (Cervantes-Castañeda et al., 2008). It has been shown that, when detected in their early (often asymptomatic) stages, the disease can be treated to slow its progression or prevent further visual impairment (Lombardo, Serrao, & Lombardo, 2022; Nagai et al., 2020). However, early detection is challenging because current gold standard ocular imaging methods such as optical coherence tomography are typically limited to detecting drusen and other major structural defects that occur in more advanced AMD (Gerson, 2017; Neely et al., 2017). Techniques relying on the perception of uniformly polarized light through the Haidinger’s brush to detect the earliest signs of AMD have historically found limited success owing to low visibility and lack of interpretable thresholds (Misson & Anderson, 2017; Müller et al., 2016; Temple et al., 2015; Temple, Roberts, & Misson, 2019).

Here, we present a novel method to quantify macular health through the selective characterization of the coMP optical density (coMPOD). The method relies on the perceptual threshold measurements of several structured light stimuli with different spatiotemporal frequencies. A study was performed with healthy young participants and found that the coMPOD is inversely proportional to retinal eccentricity in the region 1.5° to 5.5°. The presented techniques expand the capabilities of other psychophysical tests that have been developed to characterize polarization sensitivity. For example, Temple et al.  (Müller et al., 2016; Temple et al., 2019) correlated the Haidinger’s brush degree of polarization threshold to MP optical density. By incorporating additional degrees of freedom our techniques are selectively sensitive to coMP rather than total MP, providing a unique characterization tool for macular health. Potential applications include the detection of early symptoms of a variety of diseases related to macular deterioration, such as pathological myopia (Ueta et al., 2020), macular telangiectasia (Issa et al., 2013), and AMD (Thomas, Mirza, & Gill, 2021).

## Methods

Twenty-four participants with healthy retinas were recruited for the study. The study protocols conformed with the Declaration of Helsinki and each participant provided informed consent before the study. Ocular examinations, which included assessments of unaided logarithm of the minimum angle of resolution distance visual acuity, binocular vision, subjective refraction, ocular motility, slit-lamp biomicroscopy, indirect opthalmoscopy, and color fundus photography, were performed on each participant during screening. Participants with a previous diagnosis of ocular, systemic, or neurological disorders were excluded. All research procedures received approval from the University of Waterloo Office of Research Ethics.

Participants were asked to perform a psychophysical rotation direction discrimination task. The rotation is necessary because a static polarization-related entoptic phenomena disappears after a few seconds due to visual adaptation (Horváth & Varju, 2004). The setup that prepares a visual stimulus inducing an entoptic phenomena with variable azimuthal fringes and arbitrary occlusions is described in Kapahi et al. (2024): The light from a 450-nm diode laser is spatially filtered and polarized before directed onto a spatial light modulator (SLM) that is capable of imprinting arbitrary phase profiles. We used the HOLOEYE GAEA-2 SLM with temporal resolution of 60 Hz to create structured light states with radially symmetric polarization profiles possessing different numbers of azimuthal fringes. Finally, a set of lenses was used to project the state onto the retina. The SLM calibration curve (voltage vs phase shift) was experimentally determined for our wavelength using a polarimetry measurement, and the accuracy of the programmed structured images were confirmed by taking polarization measurements needed to determine the four Stokes parameters and characterize the polarization profiles of the beams. Images were also taken before each session to ensure the absence of artifacts due to dust or misalignment.

In this study, stimuli with *N* = 3, 8, 11, 13, and 18 azimuthal fringes were generated. Aligned to the center of the stimuli a 50-µm pinhole was illuminated by a 632-nm laser to generate an approximately Gaussian guide light with a maximum retinal eccentricity of 0.5° (1.0° visual angle) that participants were directed to fixate on. The participant's head position was stabilized using a head and chin rest that was placed in front of the setup to ensure a robust determination of retinal subtense. Each trial began with the presentation of a static stimulus to ensure visibility, then upon the participant’s signal, the rotating (either clockwise or counter clockwise) stimulus was presented for 500 ms. After the rotating stimulus was presented, the participant indicated the direction of rotation.

**Figure 1.**
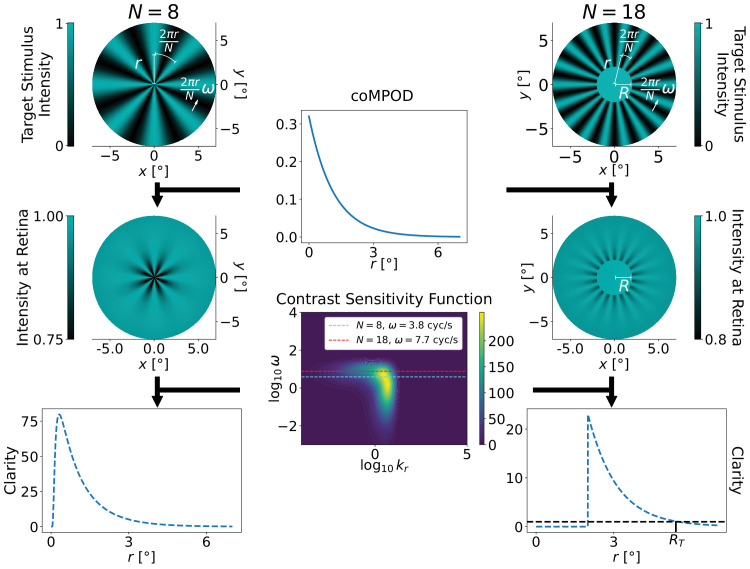
In this work, we characterize the observer’s coMP profile in the range of 1.5° to 5.5° retinal eccentricity by measuring the perceptual thresholds of a set of stimuli with unique spatiotemporal frequencies. The target stimulus consists of a central obstruction with radius *R* and *N* azimuthal fringes rotating with temporal frequency ω. The top row shows two examples of an intensity pattern where an ideal radial polarizer is considered. The efficiency of the radial polarizer in the human eye is determined by the amount of coMP, which decays with retinal eccentricity. It follows that the contrast of the pattern that a person perceives is greatest near the center. To determine a person’s ability to perceive a moving pattern we account for the viewer’s contrast sensitivity, given by the *M*-scaled contrast sensitivity function (CSF_M_[*r*, *k*_*r*_, ω]), which is equal to the inverse of the contrast threshold required for perceiving a particular spatial frequency (*k*_*r*_) and temporal frequency (ω) at retinal eccentricity (*r*). Therefore, the spatially dependent clarity (*C*[*r*]) of the observed patterns is obtained by multiplying the azimuthal contrast of the stimulus at each radius *r* by its corresponding value in the CSF_M_. The partially blocked stimulus is considered resolvable for all obstructions with radii *R* ⩽ *R*_*T*_ where *C*(*R*_*T*_) = 1.

**Figure 2.**
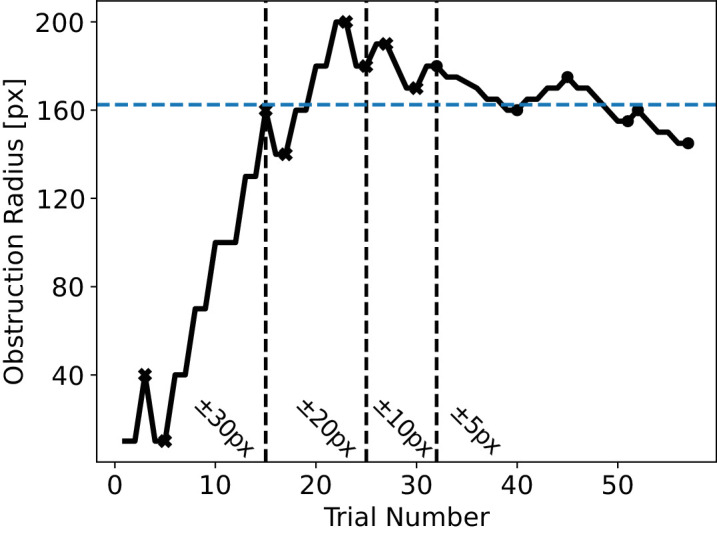
Example of a two-up/one-down staircase protocol. The reversal points are marked with black points and crosses for clarity, and the change in the obstruction size with each trial is noted in each region. Ten pixels on the SLM is approximately 0.45° retinal eccentricity. After 14 staircase reversals or 90 trials, the arithmetic mean of the final 6 reversals (marked by black points) is taken to be the participant’s threshold radius (blue dashed line) for the given stimulus.

To measure the retinal eccentricity thresholds a circular obstruction with variable size was centered on the fixation point (Figure 1). The preparation of these structured light states was accomplished via an SLM whose pixels can be individually addressed to set arbitrary spatial polarization states (Pushin et al., 2023). Before taking measurements, the participants performed a familiarization task. In this task, the initial obstruction radius was set to roughly 0.45° and the stimulus was shown for several seconds a total of 10 times. The task was repeated until each participant achieved at least 70% discrimination accuracy. After the initial familiarization, the radius of the obstruction, *R*, was changed according to the two-up/one-down staircase method described in Levitt (1971): two consecutive correct answers were required to increase the radius of the obstruction, and each incorrect answer resulted in a decrease. This method allows for a 70.7% performance accuracy measurement of the threshold radius for each stimulus. Figure 2 shows an example of the staircase for one of the participants. Each measurement was ended after either 14 staircase reversals (i.e., the radius of the obstruction changes from increasing to decreasing or vice versa) or 90 total trials. Initially, the obstruction was set to a radius of approximately 0.45°. The step size of the change in the central obstruction radius in visual degrees was 30 pixels (approximately 1.35°) up to the third reversal, then 20 pixels (approximately 0.90°) up to the sixth, 10 pixels (approximately 0.45°) up to the ninth, and 5 pixels (approximately 0.225°) after nine reversals. Furthermore, the participant was randomly at a rate of 10% shown a stimulus with an obstruction radius either 10 pixels (approximately 0.45°) or 30 pixels (approximately 1.35°), whose results were not considered. Note that the subjective retinal eccentricity to pixel size conversion values were determined using the structured light imaging method of Kapahi et al. (2024). The final threshold radius was calculated as the arithmetic mean of the final six reversal points. If the participant completed the maximum number of 90 trials, the final point was treated as a reversal point.

To ensure a robust result, we considered the participants who were extremely sensitive to the stimuli. Any participant whose results contained two or more reversals at the minimum obstruction radius within the final six reversals was assigned a failed status for that particular data point, which was removed from further analyses. Further, any data point lying outside a 99.9% confidence interval (±3.3 σ) was excluded. After failures and outliers were removed, any participant with two or fewer retinal eccentricity threshold values remaining were also excluded because their results could not be reliably modelled. In total, 15 participants were included in the final analysis. We imposed these strict inclusion criteria because the number of data points taken for each participant was relatively low over a large range of threshold radii.

## Model

Figure 1 outlines the developed model for reconstructing coMPOD profiles using retinal eccentricity thresholds for structured light stimuli. The target stimulus is a radially symmetric polarization profile with *N* fringes and rotating with temporal frequency ω in Herz, corresponding with an angular velocity 2πω/*N*. The stimulus passes through a participant’s cornea and MP before perception. The cornea induces birefringence, and a portion of total MP, which is circularly oriented, acts as a weak radial polarizer. The perceived intensity is given by:

(1) $$\begin{array}{ccc} & & \;I\left(r,\phi ,t\right)=\frac{1}{4\pi \sigma_{p}^{2}}e^{\frac{-r^{2}}{\sigma_{p}^{2}}}(4+\Theta \left[r-R\right]P\left(r\right)\\ & & \;[\cos(N\phi +2\pi \omega t)+\cos((4+N)\phi +2\pi \omega t)\\ & & \;+2\cos\beta \sin2\phi \sin((2+N)\phi +2\pi \omega t)-2]),\; \end{array}$$

where *R* is the radius of the central obstruction, σ_*p*_ is the beam width, (*r*, ϕ) are retinal eccentricity coordinates, *t* is time, Θ[*r*] is the Heaviside step function, β is the corneal birefringence-induced phase shift, and *P*(*r*) is the efficiency of the radial filter arising from coMP. For negligible corneal birefringence or in cases where corneal birefringence is compensated for, β ≈ 0 and the equation for the perceived intensity simplifies to:

(2) $$\begin{array}{ccc} & & I\left(r,\phi ,t\right)=\frac{e^{\frac{-r^{2}}{\sigma_{p}^{2}}}}{2\pi \sigma_{p}^{2}}(2+\Theta \left[r-R\right]P\left(r\right)\\ & & \;\times \;\left[\cos(N\phi +2\pi \omega t)-1\right]). \end{array}$$

See the Supplementary Material of Kapahi et al. (2024) for corneal birefringence considerations. The efficiency of the radial polarizer in the human eye can be modelled by:

(3) $$P\left(r\right)=1-10^{-M(r)},$$

where M(*r*) is the profile of the coMPOD.

To determine if an individual can resolve the fringes of the stimulus, we first calculate the radially varying contrast *V*(*r*). For a given radial fringe pattern *f*(ϕ, *t*) = *A* + *B*cos (*k*_ϕ_ϕ − *k*_*t*_*t*), the contrast is time invariant and defined as *V* = *B*/*A*.

The contrast sensitivity function describes the lowest perceivable contrast *V*_*T*_(*k*_*r*_, *k*_*t*_) for different spatial and temporal frequencies (van den Branden Lambrecht, 2001):

(4) $$\mathrm{CSF}\left(k_{r},k_{t}\right)=\frac{1}{V_{T}\left(k_{r},k_{t}\right)}.$$

*V*_*T*_(*k*_*r*_, *k*_*t*_) has been experimentally determined for linear fringe patterns presented to the center of the visual field. To account for the effects of cortical magnification on contrast sensitivity outside the fovea, we can transform the given on-axis contrast sensitivity function to an equivalent representation outside central vision through *M*-scaling (Daniel & Whitteridge, 1961; Rovamo & Virsu, 1979; Strasburger, Rentschler, & Jüttner, 2011; Virsu, Rovamo, Laurinen, & Näsänen, 1982). To construct the *M*-scaled contrast sensitivity function, CSF_M_(*r*, *k*_*r*_, *k*_*t*_), any spatial frequency from the on-axis CSF is transformed according to *k* → *k*/*s*(*r*). Here *s*(*r*) ≈ (1 + 0.42*r*)^−1^ is dimensionless, such that defining *M*_0_ to be the cortical magnification at *r* = 0, *s*(*r*) · *M*_0_ describes the projection of the visual field onto the visual cortex in dimensions of millimeters per visual degree.

We can then define clarity, *C*(*r*), to be the ratio of the stimulus contrast and the lowest perceivable contrast:

(5) $$C\left(r\right)\equiv V\left(r\right)\cdot {\mathrm{CSF}}_{M}\left(r,\frac{N}{2\pi r},\omega \right)$$

and the peak clarity of the stimulus:

(6) $$C_{P}\equiv \max\left[C\left(r\right)\right].$$

By definition, if *C*_*P*_ < 1 the direction of the pattern’s rotation is indiscernible. Since the efficiency of the eye’s radially polarized light filter decays quickly with *r* in the region of interest, the pattern that an individual will perceive is sharpest near the center and contrast quickly decays as *r* increases. Owing to this decreasing nature of *C*(*r*), the radius *r* = *R*_*T*_ at which *C*(*R*_*T*_) = 1 defines the retinal eccentricity threshold of the particular stimulus. Note that this approach is geared toward characterizing circularly oriented MP that is decreasing with retinal eccentricity. To characterize any asymmetries in the profile we would need to incorporate outer obstructions in the stimuli in addition to inner obstructions.

## Results and discussion

The presented technique is selectively sensitive to coMPOD, rather than total MPOD. The profile of the total MPOD has been studied elsewhere (Berendschot & van Norren, 2006), and the analysis assuming a similar profile for the coMPOD is presented in the Appendix. These results and the geometry of the Henle fiber layer motivate modelling the coMPOD in the range of 1.5° to 5.5° retinal eccentricity by:

(7) M(r)=a/(2πr),

where *a* is a constant. Equation 7 assumes that the total number of Henle’s fibers and the MP they contain does not vary on average in the region of interest; all that occurs is that these fibers spread out with eccentricity as more space becomes available. The M(*r*) in Equation 7 is, therefore, proportional to the density of fibers at radius *r*; the factor *a* is influenced by the number of Henle’s fibers as well as the efficiency of the radial polarization effect of the contained MP. Note that this form for the coMPOD is not suited for the low eccentricity region. coMPOD must be bound according to coMPOD → 0 as *r* → 0. This implies some maximum optical density is reached near the center of the fovea before the decay at the very center.

**Figure 3.**
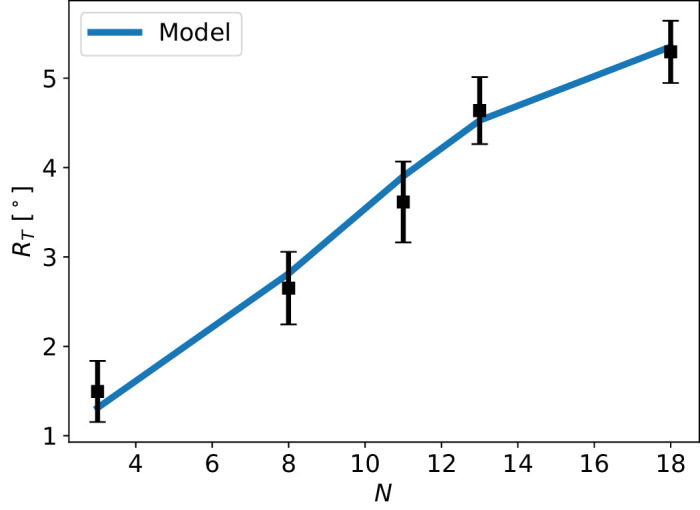
The average of the retinal eccentricity threshold results for the five different stimuli presented in this study: (*N* = 3, ω = 1.9 Hz), (*N* = 8, ω = 3.8 Hz), (*N* = 11, ω = 5 Hz), (*N* = 13, ω = 5.8 Hz), and (*N* = 18, ω = 7.7 Hz). Also shown are the averages of threshold radii obtained through fitting the coMPOD model for individual participants. The Pearson χ^2^ fit statistic comparing the coMPOD model and the data is approximately 0.06, indicating a good fit.

**Figure 4.**
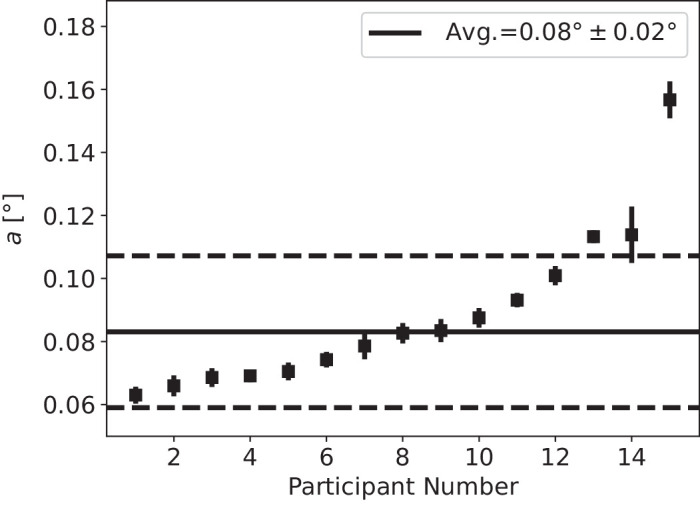
The obtained coMPOD parameter for each individual participant. The average *a* was found to be $\bar{a}=0.08{}^{\circ }\pm 0.02{}^{\circ }$. Shown in dashed lines are ±1 standard deviation. Error bars on each data point denote an approximate 99.9% confidence interval.

Figure 3 shows that the model can fit the average threshold values well; the Pearson χ^2^ fit statistic is approximately 0.06 with 4 degrees of freedom. Figure 4 shows the individual participant fits for the parameter *a* where the average *a* was found to be $\bar{a}=0.08{}^{\circ }\pm 0.02{}^{\circ }$. A comparison between this average profile for coMPOD and the average profile for total MPOD (Berendschot & van Norren, 2006) reveals that a small portion (approximately 1/10) of total MP contributes to coMP for the low eccentricity values near 1.5°, whereas at higher values of approximately 5.5° they are roughly equal.

Recent studies have increasingly explored the relationship between macular structure and the perception of Haidinger's brush (Müller et al., 2016; Temple et al., 2019), modelling the effective radial polarizer in the macula (Misson, Temple, & Anderson, 2019; Müller et al., 2016; Wang, Bryanston-Cross, Timmerman, Li, & Liu, 2020), and developing contrast sensitivity models for human polarization perception (Wang, Bryanston-Cross, Timmerman, Li, & Liu, 2022a; Wang, Bryanston-Cross, Timmerman, Li, & Liu, 2022b). Researchers have also experimented with alternative polarization patterns (Kapahi et al., 2024; Misson & Anderson, 2017; Sarenac et al., 2022) to enhance the inherently weak signals of Haidinger's brush (O’Shea, Misson, & Temple, 2021). A key motivation behind these efforts is the potential for a novel screening technique for degenerative macular diseases like AMD. Techniques have been developed to quantify human sensitivity to polarized light by measuring the lowest degree of polarization at which Haidinger's brush is perceived (Temple et al., 2015), with findings showing reduced polarization sensitivity in AMD patients (Misson, Anderson, Armstrong, Gilett, & Reynolds, 2021).

The methods introduced here build on previous work to provide the first direct characterization of biological elements, namely, circularly oriented MP, that are responsible for human perception of polarized light. In all previous research involving human perception of polarized light, as well as other studies evaluating the progression of macular degeneration (Raman, Biswas, Vaitheeswaran, & Sharma, 2012; Tsika et al., 2011), MPOD was associated with macular health. However, recent studies have shown that patients with early and intermediate age-related macular degeneration (AMD) may exhibit higher MPOD than age-matched controls (Kar et al., 2020; McGwin Jr et al., 2023). Our work not only addresses these seemingly contradictory findings by differentiating between MPOD and coMPOD, but also opens up new possibilities for future research and experimentation.

## Conclusions

We have demonstrated a novel method to selectively characterize the coMPOD, thereby providing a procedure to quantify the microstructure of the macula for applications in the diagnosis of macular disease. Future work will consider a direct comparison with total MPOD measurements via autofluorescence. This would provide a general ratio between MPOD and coMPOD. To actualize a clean demonstration for specifically measuring coMPOD, the probed retinal eccentricity region in this study was 1.5° to 5.5° owing to the generally well-behaved exponential decay of MPOD. Future studies will also probe the coMPOD at lower eccentricities, particularly the 0° to 1.5° region, where some distortions in vision and variable spatial distributions of MPOD have been reported (Rowland & Lee, 2019).

Early treatment of AMD and other macular diseases is crucial to minimizing or eliminating visual impairment. Therefore, a more selective method of early diagnosis would improve patient prognoses. The presented techniques can be applied to a wide range of stimuli and macular defects. Specific use cases could include those of the early diagnoses of AMD (Thomas, Mirza, & Gill, 2021), pathological myopia manifesting as a tear in the macula (Ueta et al., 2020), and macular telangiectasia, which is often associated with changes in the MP (Issa et al., 2013).

## Figures and Tables

**Figure 5.**
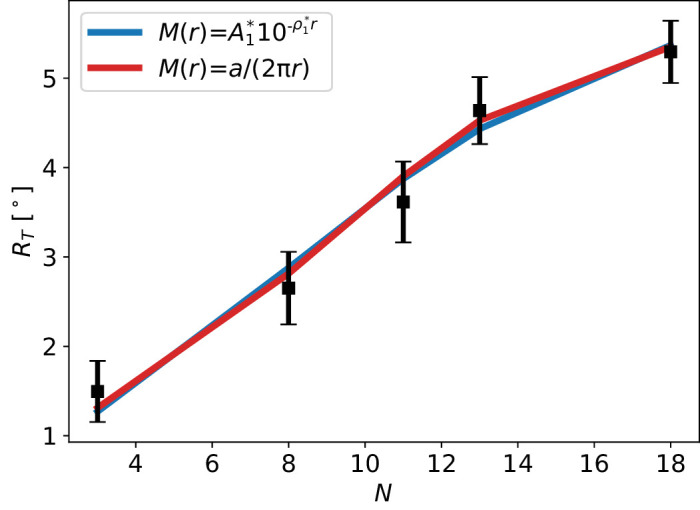
The average of the retinal eccentricity threshold results for the five different stimuli presented in this study: (*N* = 3, ω = 1.9 Hz), (*N* = 8, ω = 3.8 Hz), (*N* = 11, ω = 5 Hz), (*N* = 13, ω = 5.8 Hz), and (*N* = 18, ω = 7.7 Hz). Also shown are the averages of threshold radii obtained through fitting Equation 11 (blue line) and Equation 7 (red line) for individual participants. The Pearson χ^2^ fit statistic for Equation 11 is approximately 0.09 (with 3 degrees of freedom). That of Equation 7 is approximately 0.06 (with 4 degrees of freedom), indicating that both are good fits, though the Equation 7 fit is significantly more robust as it considers one fit parameter rather than two fit parameters.

**Figure 6.**
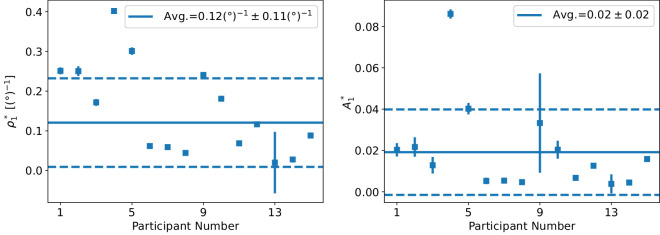
The obtained coMPOD parameters for each individual participant and the average using Equation 11. The average parameters were found to be $\bar{\rho_{1}^{*}}=0.12{\left({}^{\circ }\right)}^{-1}\pm 0.11{\left({}^{\circ }\right)}^{-1}$, and ${\bar{A}}_{1}^{*}=0.02\pm 0.02$. The average $A_{1}^{*}$ being significantly lower than the average value of *A*_1_ (peak total MPOD) in Berendschot and van Norren (2006) implies that coMP is a small portion of total MP. Uncertainties in the fits are significantly more varied, as the absence of data points in some participants has a greater effect when fitting two parameters. Shown in dashed lines are ±1 standard deviation. Data is ordered according to Figure 4 in the main article. Error bars on each data point denote an approximate 99.9% confidence interval.

## Appendix

### MPOD profile model

The efficiency of the radial polarizer in the human eye can be modelled by:

(8) $$\begin{array}{c} P\left(r\right)=1-10^{-M(r)},\; \end{array}$$

where M(*r*) is the coMPOD. Here we assume that M(*r*) is described by an equation that has a similar form as the total MPOD function *m*(*r*) (Berendschot & van Norren, 2006):

(9) $$\begin{array}{c} m\left(r\right)=A_{1}10^{-\rho_{1}r}+A_{2}10^{-\rho_{2}{\left(r-\alpha_{2}\right)}^{2}},\; \end{array}$$

where *A*_1_ is the peak MPOD (at the fovea), ρ_1_ describes the slope of the MPOD function, and *A*_2_, ρ_2_, and α_2_ describing the amplitude, slope, and location of the secondary ring. The average parameters for an aged population (50 ± 17 years old) have been measured to be *A*_1_ = 0.31 ± 0.12, ρ_1_ = 0.38(°)^−1^ ± 0.24(°)^−1^, *A*_2_ = 0.11 ± 0.08, ρ_2_ = 1.2(°)^−2^ ± 1.1(°)^−2^, and α_2_ = 0.70° ± 0.66° (Berendschot & van Norren, 2006). In the regime of *r* this study investigated, the second term becomes negligible with typical parameters, so the MPOD function can be well-approximated by:

(10) $$\begin{array}{c} m\left(r\right)\approx A_{1}10^{-\rho_{1}r},\; \end{array}$$

Therefore, we take the coMPOD profile to be:

(11) $$\begin{array}{c} M\left(r\right)=A_{1}^{*}10^{-\rho_{1}^{*}r}\; \end{array}$$

where $A_{1}^{*}$ is the peak coMPOD and $\rho_{1}^{*}$ describes the slope.

Figure 5 shows the average values of *R*_*T*_ for study participants, as well as averages of *R*_*T*_ fit to individuals’ data using MPOD profile of Equation 11, for the free parameters $\rho_{1}^{*}$ and $A_{1}^{*}$. Comparing the two sets of values yields a Pearson goodness of fit statistic of 0.09 on 3 degrees of freedom, showing good agreement between the data set and the model fits. Also shown on Figure 5 for comparison is the fit presented in the manuscript with M(*r*) = *a*/(2π*r*).

Figure 6 shows the fitted values of $\rho_{1}^{*}$ and $A_{1}^{*}$ for each participant. These values were used for modelling the average values of *R*_*T*_ as shown in Figure 5. We find that the average value of individuals’ $\rho_{1}^{*}$ is 0.12(°)^−1^ ± 0.11(°)^−1^, and the average of individuals’ $A_{1}^{*}$ is approximately 0.02 ± 0.02, which is significantly lower than the peak MPOD found in Berendschot and van Norren (2006). Noting the approximation *A*10^−.14*r*^ ≈ *A*/*r* for *r* ∈ {1.5, 5.5}, motivates the use of the coMPOD form as described in the article. The latter is significantly more robust because it considers one fit parameter rather than two fit parameters.
